# Environmental surveillance of commonly-grown vegetables for investigating potential lead and chromium contamination intensification in Bangladesh

**DOI:** 10.1186/s40064-016-3458-9

**Published:** 2016-10-18

**Authors:** A. M. M. Maruf Hossain, M. Shahidul Islam, M. Mustafa Mamun, H. M. Al-Jonaed, M. Imran, M. Hasibur Rahman, M. Azizul Islam Kazi, Syed Fazle Elahi

**Affiliations:** 1 Department of Soil, Water and Environment, Faculty of Biological Sciences, University of Dhaka, Dhaka, 1000 Bangladesh; 2 Analytical Research Division, BCSIR Laboratories, Dr. Qudrat-i-Khuda Road, Dhanmondi, Dhaka, 1205 Bangladesh; 3 School of Global, Urban and Social Studies, RMIT University, Melbourne, VIC 3000 Australia

**Keywords:** *Heavy Metal* contamination, Vegetables, Lead, Chromium, Cadmium, Environmental public health assessment, Semi-quantitative analysis, Bangladesh

## Abstract

With regard to previously reported Lead (Pb) and Chromium (Cr) contamination in egg, a semi-quantitative assessment of the general environment of Bangladesh is carried out through nation-wide sampling of commonly grown and consumed vegetables. Five vegetables, namely, White Potato (*Solanum tuberosum*), Green Cabbage (*Brassica oleracea capitata* var. *alba* L.), Red Spinach (*Amaranthus dubius*), White Radish (*Raphanus sativus* var. *longipinnatus*), and Green Bean (*Phaseolus vulgaris*) were selected for sampling from all 64 Districts of the country as per their availability during the sampling season. This results in a collection of 292 samples. Due to the necessity of incorporating a wide spectrum of vegetable types (e.g. tuber, brassica, leafy, root, and fruiting vegetables) as well as the need for including the geographic expanse of the whole country, replicates were not accommodated in the study, hence, the study being semi-quantitative in nature. The results do not substantiate evidence of public health risk due to Cr yet, with only 0.69 % of the samples found contaminated with Cr. Pb contamination (concentrations above a health-based guidance value) is found in 29.47 % of the samples; and together with the insight of such contamination likely being non-point origin, communicates on potential public health risk due to Pb as residual effects of extensive ambient atmospheric Pb pollution in recent history of the country. For the purpose of comparison, Cadmium (Cd) contents of the samples are also analyzed. Although there is no extensive Cd pollution history/source in the country, the likely point-source nature of Cd contamination found in 17.83 % of the samples indicates the scope of managing any existing contamination source by directing efforts into the potential point-sources.

**Electronic supplementary material:**

The online version of this article (doi:10.1186/s40064-016-3458-9) contains supplementary material, which is available to authorized users.

## Background

In recent years, researches report on Pb (Lead) and Cr (Chromium) contamination in pountry egg in Bangladesh. Hossain et al. (2010) reports that in poultry egg, sampled from eight Districts of central Bangladesh (including the capital Dhaka), the dry weight basis mean Pb concentration was found to be 8.1611 ppm, which is about 20 times higher than the maximum permissible limit when compared with dry weight basis maximum permissible limit [converted from fresh weight basis maximum permissible limit of 0.1 µg/g set by FAO/WHO (2006)]. Such a phenomenon was suspected with possible ecotoxicological fate of recent extensive ambient atmospheric Pb pollution in the country (Hossain et al. 2010), the residual effects of which was already detected in human body such as blood Pb levels in school-going children (Kaiser et al. 2001). The Pb pollution in poultry egg has added significance due to the wide practice of use of locally grown feed ingredients, manually incorporated into poultry feeds.

From similar study, Hossain et al. (2009) also reported on potential Cr contamination of eggs, having a dry weight basis mean Cr concentration of 1.9016 ppm, thus, an egg containing mean chromium content of 23.3809 μg, which exceeds adequate daily dietary intake of children up to 8 years of age [up to 15 μg/day (Institute of Medicine 2001)] as well as corresponds to major part for other age groups. The case of Cr contamination had a suspected highly particular route with the use of chrome-tanned skin cut solid waste converted into protein-concentrates that are highly Cr-contaminated, for use in poultry feeds, fish feeds and agricultural application as organic fertilizer (Hossain et al. 2007).

Given this background, this study undertakes a semi-quantitative assessment of the general environment of the country for Pb and Cr contamination through conducting nation-wide surveillance of five locally-grown common vegetables. The levels of Cadmium (Cd), being another food contaminant as *heavy metal*, were also analyzed for the purpose of comparing with Pb and Cr contamination status of the samples. This study, therefore, serves two objectives: (1) semi-quantitatively assessing the general environment of Bangladesh due to the intimate linkage through soil–water–air continuum with agricultural products, as well as, (2) informing public health safety from these five commonly grown and consumed vegetables.

## Methods

### Sampling

Bangladesh consists of 64 Districts. Five vegetables commonly grown and consumed throughout the country were selected for sampling, these being: (1) White Potato (*Solanum tuberosum*), (2) Green Cabbage (*Brassica oleracea capitata* var. *alba* L.), (3) Red Spinach (*Amaranthus dubius*), (4) White Radish (*Raphanus sativus* var. *longipinnatus*), and (5) Green Bean (*Phaseolus vulgaris*). The vegetables correspond to five broad vegetable groups, White Potato (#1) falling under ‘tuber vegetable’, Green Cabbage (#2) under ‘brassica vegetable’, Red Spinach (#3) as ‘leafy vegetable’, White Radish (#4) under ‘root vegetable’, and Green Bean (#5) being from the ‘fruiting vegetable’ group. From among the 64 Districts of the country, all vegetable types were sampled from 36 Districts, only one and two vegetable types respectively were found available in two Districts, and at least three to four vegetable types were sampled from the remainder 26 Districts. From the capital Dhaka and Rangpur District, sampling was conducted from two distinct Thanas (sub-district). In some Districts, alternate vegetable types were sampled from different Thanas due to varying availability throughout the district, which were recorded accordingly. Thus, the nation-wide sampling had produced a total count of 292 samples.

Due to multiple reasons relating to the necessity of incorporating a wide spectrum of vegetable types as well as inclusion of the entire geographical expanse of the country, replicates could not have been accommodated in this study, hence, the study being semi-quantitative in nature. The reason behind choosing five vegetables was not merely for increasing the sample diversity; rather to impart a type of diversity that would provide self-sufficiency to the assessment. As the five vegetables cover a broad spectrum of tuber, brassica, leafy, root, and fruiting vegetables, this enables testing samples from a wide variety of plant organs to deduce contamination scenario through the soil–water–air continuum in the biogeochemical cycle. Besides, the vegetables selected from these five categories (one vegetable from each category) are widely consumed as well as grown throughout the country. As there are particular contamination routes throughout the country with regard to Pb and Cr, therefore, it is also necessary to incorporate the entire geographical expanse of the country in order to reveal the ecotoxicological fate of recent extensive ambient atmospheric Pb pollution in the country, as well as the impact of the Cr contamination phenomenon through wide dissemination of protein-concentrates produced from chrome-tanned skin cut solid waste for farming practice. It is the inclusion of such wide spectrum of vegetable types as well as the entire expanse of the country into the research design that provides the true significance of the study. Due to these necessities, the study has been conducted in semi-quantitative nature, with an already extensive sample base.

The details of sampling (District alphabetical IDs and names as well as the Thana(s) as sample source under each District) are provided in Additional file 1: Table S1. As the *heavy metal* contamination standards for foods are available in fresh weight basis, the standard moisture content for each vegetable is taken to convert the fresh weight basis standards into corresponding dry weight basis measures, so that the results generated in dry weight basis measure could be compared to the standards. This conversion is accomplished by determining how many grams of fresh weight sample correspond to per gram dry weight sample (based on the respective moisture content of the vegetables), and thereafter multiplying the fresh weight basis standard that many times to derive the corresponding dry weight basis standard. The standard moisture contents reported by the University of Kentucky (1997) for White Potato, Green Cabbage, Red Spinach and White Radish are 79, 93, 92, and 95 % respectively; while the reported moisture content (Doymaz 2011) for Green Bean is 89.5 %. The general equation for determining these moisture contents is:

$$\left[ {\% \,{\text{Moisture}} = \left( {m_{\text{w}} /m_{\text{sample}} } \right) \times 100} \right],$$

where *m*_w_ stands for mass of water in the sample, and *m*_sample_ stands for mass of the sample.

### Sample pretreatment, preparation and analysis

The unpeeled samples were pretreated through thoroughly washing with tap-water to clean any dirt attached to the surface, followed by carefully washing with distilled water before they were cut and sliced for oven drying. Deionized water could not have been used due to requirement in large quantities. The samples (20 g fresh weight from each) were then oven-dried at 80 °C in order to remove all moisture. The oven-drying was repeated until the difference between two subsequent readings became negligible, which took between 8 to 12 h.

The samples (0.2 g dry weight from each) were prepared through HNO_3_–HClO_4_ digestion (Kebbekus and Mitra 1998). Being organic origin as the samples have a very high organic content, the HNO_3_–HClO_4_ digestion was preferred over the more common HNO_3_ extraction for the determination of *heavy metals*. This strongly oxidizing digestion decomposes organics quickly and efficiently.

The Pb, Cr, and Cd concentrations were analyzed in atomic absorption spectrophotometry (AAS), with BDH (British Drug Houses) standards utilized in preparing the calibration curves. The measurements were conducted in air-acetylene flame AAS. The minimum detection limits were at parts per billion (ppb) level for all three *heavy metals* for the 0.2 g dry weight sample digested into 100 ml volume. This is quite dilute concentration compared to 1 g of sample dry weight (for the concentrations to be expressed per gram dry weight). After calibrating the sample readings (by subtracting the blank reading from the sample readings) when the calibrated readings reached 0 ppb, those samples were taken at undetected level for the given *heavy metal*. Where the calibrated readings produced some value at ppb level, these were further converted into dry weight basis μg/g levels. This is how the ND level was determined (ND = not detected, at ppb level for 0.2 g dry sample digested into 100 ml volume) and the detected levels expressed at ppm level (μg/g dry weight basis).

## Results and discussion

As the sampled vegetables correspond to diverse vegetable groups and the contaminants analyzed from the samples are also not evenly concerned in the literature in terms of contamination standards, it becomes necessary to construct a scheme of standards for the contaminants for these vegetable types. As Pb and Cd are classified as food contaminants, the standards for their maximum safe levels in foods have been proposed in the literature. However, as Cr is considered as a required mineral for humans in trace quantities (NRC 1980; Mertz 1998), this imparts complexity in proposing contamination standards for Cr. The dietary reference intakes (DRI) for Cr was established in 2001, where due to insufficient research-base for establishing RDAs (recommended dietary allowances), AIs (adequate intakes) were proposed instead (Institute of Medicine 2001). However, the tolerable upper intake levels (UL) in terms of AIs were not established due to the existence of evidence for adverse effects linked with high Cr intakes (Stoecker 2001; Institute of Medicine 2001). Given this reality, the USEPA IRIS (U.S. Environmental Protection Agency, Integrated Risk Information System) chronic oral Reference Dose (RfD) for Cr(VI) is compared as a conservative approach in assessing the hazard potential of the Cr contents of the samples. Although the RfD for Cr(VI) represent a more toxic state of Cr compared to the other Cr oxidation states, in the scheme of oral RfD for Cr, Cr(VI) represents the lowest safe threshold in terms of inducing toxicity. For example, the USEPA IRIS oral RfD for Cr(III) is 1.5 mg/kg-day (USEPA, 1998a), whereas the same for Cr(VI) becomes 0.003 mg/kg-day (USEPA, 1998b). Converting these for a 70 kg adult person comes to a threshold of 105,000 μg/70 kg-day for Cr(III), while 210 μg/70 kg-day for Cr(VI). Thus, Cr(VI) oral RfD represent a conservative approach in comparing the hazard potential in the absence of direct Cr standards for foods. The Cr content in 50 g fresh weight sample (through modeling 50 g fresh weight vegetable consumption for an adult per day) is compared against this Cr(VI) oral RfD of 210 μg/70 kg-day in order to assess potential Cr hazard.

In the cases of Pb and Cd, the maximum permissible levels for the vegetable types are reported in fresh-weigh-basis standards (FAO/WHO 2006). Converting such fresh-weight-basis standards into dry weight basis measures (through taking the standard moisture contents into consideration as described in “Sampling” section) provides the scheme of standards for the *heavy metals* in Table 1 for the vegetable types sampled in this work.

**Table 1:** Scheme of standards for contamination comparison

Sample serial	Sample name	Moisture (%)	Vegetable type	FW standard for Pb (µg/g)	DW standard for Pb (µg/g)	Standard for Cr (µg Cr per 50 g FW consumption)	FW standard for Cd (µg/g)	DW standard for Cd (µg/g)
1	White Potato	79	Tuber vegetable	0.1	0.47619	210 μg/70 kg-day	0.1	0.47619
2	Green Cabbage	93	Brassica vegetable	0.3	4.28571		0.05	0.71429
3	Red Spinach	92	Leafy vegetable	0.3	3.75		0.2	2.5
4	White Radish	95	Root vegetable	0.1	2		0.1	2
5	Green Bean	89.5	Fruiting vegetable	0.1	0.95238		0.05	0.47619

*FW* fresh weight, *DW* dry weight

In Table 1, the Pb and Cd fresh-weight-basis standards are adopted from FAO/WHO (2006), whereas a vegetable sample containing more than 210 μg/70 kg-day Cr per 50 g fresh weight consumption is considered as contaminated. Due to the extensive length of the detail results combining each sample’s source, results of sample analysis, comparison with standards, and comments on the *heavy metals*’ status, these are provided in Additional file 1: Table S1. Instead, the core findings from the results are reported and discussed here.

The overall statistics of the detail results are presented in Table 2. In the table, the minimum detected level for Cr (in terms of Cr concentration) is not reported as the comparison with Cr standard is provided on the basis of total Cr content in 50 g fresh weight of the vegetables. Similarly, in Additional file 1: Table S1 samples with ND level (Not Detected, at ppb level for 0.2 g dry sample digested into 100 ml volume) are designated as ‘undetermined’ while reporting the Cr content in 50 g fresh weight of sample.

**Table 2:** Overall statistics of the data for the metals across all vegetables

*Heavy metal*	Total count of samples analyzed	Contamination found in samples			Samples with safe levels			Samples yielding ND			Median, µg/g dry weight basis	Upper Quartile, µg/g dry weight basis
		Total count	% of total count of analyzed samples	Maximum level occurred	Total count	% of total count of analyzed samples	Minimum level detected, dry weight basis	Total count	% of total count of analyzed samples	% of total sample count with safe levels		
Pb	285	84	29.47	48.43 µg/g dry weight basis	201	70.53	0.002 µg/g	192	67.37	95.52	7.601	15.237
Cr	290	2	0.69	305.991 µg in standard 50 g FW	288	99.31	–	245	84.48	85.07	1.637	4.614
Cd	286	51	17.83	5.484 µg/g dry weight basis	235	82.17	0.049 µg/g	150	52.48	63.83	0.67	1.2678

*ND* not detected (at ppb level for 0.2 g dry sample digested into 100 ml volume), *FW* fresh weight

Table 3 presents the statistics of the detail results on Cr across the vegetable types. With regard to the potential public health risk due to Cr as referred in “Background” section, the results do not substantiate evidence of such yet. On the contrary, the percentage of the total count of samples exhibiting safe Cr levels is nearly 100 % (99.31 %, Table 2). Moreover, a vast majority of the samples (84.48 %, Table 2) yielded undetected level of Cr (not detected at ppb level for 0.2 g dry sample digested into 100 ml volume). The two samples yielding contaminated Cr levels are White Potato samples. When the Cr concentrations are compared across the five vegetable types, it is noticeable that the upper quartiles are multiple times higher than the median concentrations for all vegetable types except for Green Cabbage, while the median and upper quartiles for Green Cabbage Cr concentrations are much closely situated to each other. The existence of only two instances of Cr contamination across the total count of 290 samples throughout the entire country refers to these contamination instances most likely being point-source. Among the five vegetable types, White Potato samples have the least count of ND Cr levels (not detected, at ppb level for 0.2 g dry sample digested into 100 ml volume); whereas Red Spinach and White Radish have the highest two counts of ND levels.

**Table 3:** Statistics of the data on Cr

Vegetables	Total count of samples analyzed	Contamination found in samples			Samples with safe levels			Samples yielding ND			Median, µg/g dry weight basis	Upper Quartile, µg/g dry weight basis
		Total count	% of total count of analyzed samples	Maximum level occurred	Total count	% of total count of analyzed samples	Minimum level detected, dry weight basis	Total count	% of total count of analyzed samples	% of total sample count with safe levels		
White Potato	50	2	4	305.991 µg in standard 50 g FW	48	96	–	35	70	72.92	2.399	8.004
Green Cabbage	49	0	0	–	49	100	–	41	83.67	83.67	1.1665	2.1363
Red Spinach	61	0	0	–	61	100	–	58	95.08	95.08	0.288	3.593
White Radish	65	0	0	62.9325 µg in standard 50 g FW	65	100	–	59	90.77	90.77	2.3935	12.0765
Green Bean	65	0	0	130.9245 µg in standard 50 g FW	65	100	–	52	80	80	1.388	6.2755
Overall	290							245	84.48		1.637	4.614

*ND* not detected (at ppb level for 0.2 g dry sample digested into 100 ml volume), *FW* fresh weight

The statistics of the Cd data across the five vegetable types are listed in Table 4. Although there is no extensive Cd pollution source in Bangladesh (unlike Pb and Cr), yet the count of Cd contaminated samples found to be 51 (which mounts to 17.83 % of total sample count, Table 2) is likely to be linked to point-source Cd pollution rather than residual effect of any major ecotoxicological phenomenon. White Potato and Green Bean samples account for the most of the Cd contaminated samples in contrast to the other three vegetable types. Source-tracking of the contaminated samples reveal 4 Districts containing 3 contaminated samples each, 7 Districts with 2 contaminated samples each, whereas 24 Districts containing only 1 contaminated sample in each. The rest of the 29 Districts not containing any Cd contaminated sample further indicates on point-source nature of the Cd pollution in the samples. A statistical comparison of the Cd data across the five vegetable types reveals both of the upper quartile and median Cd concentrations to be higher than the Cd standards for White Potato and Green Bean, whereas these being lesser than the Cd standards for the rest of the three vegetable types (i.e. Green Cabbage, Red Spinach and White Radish).

**Table 4:** Statistics of the data on Cd

Vegetables	Total count of samples analyzed	Contamination found in samples			Samples with safe levels			Samples yielding ND			Median, µg/g dry weight basis	Upper Quartile, µg/g dry weight basis	Dry weight basis standard for Cd, µg/g
		Total count	% of total count of analyzed samples	Maximum level occurred	Total count	% of total count of analyzed samples	Minimum level detected, dry weight basis (µg/g)	Total count	% of total count of analyzed samples	% of total sample count with safe levels			
White Potato	49	15	30.61	5.484 µg/g dry weight basis	34	69.39	0.049	26	53.06	76.47	0.741	1.93	0.47619
Green Cabbage	49	4	8.16	1.11 µg/g dry weight basis	45	91.84	0.098	32	65.31	71.11	0.436	0.64	0.71429
Red Spinach	60	5	8.33	5.323 µg/g dry weight basis	55	91.67	0.05	27	45	49.09	0.885	1.765	2.5
White Radish	64	5	7.81	3.638 µg/g dry weight basis	59	92.19	0.149	35	54.69	59.32	0.746	1.4765	2
Green Bean	64	22	34.37	5.092 µg/g dry weight basis	42	65.63	0.095	30	46.88	71.43	0.654	1.1115	0.47619
Overall	286	51	17.83	5.484 µg/g dry weight basis	235	82.17	0.049	150	52.48	63.83	0.67	1.2678	

*ND* not detected (at ppb level for 0.2 g dry sample digested into 100 ml volume)

The statistics of data on Pb across the five vegetable types are reported in Table 5. The Pb contamination is found to be somewhat evenly distributed across the vegetable types, except for Green Cabbage exhibiting the least number of Pb contaminated samples (over 50 % less than the other four). The maximum Pb level in Green Cabbage samples is also found to be the lowest within the detected maximum range of (29.794–48.43 µg/g) (dry weight basis) across the vegetables. Source-tracking of the contaminated samples reveal that out of the 84 contaminated samples 2 Districts contained 4 contaminated samples each, 5 Districts containing 3 contaminated samples each, whereas 1 and 2 contaminated samples corresponding to 24 and 18 Districts, respectively. Only the remaining 15 Districts (out of the 64) did not produce any Pb contaminated samples. This portrays a distributed presence of Pb contamination in the vegetables across the country, detected in approximately 29.47 % of the total sample count (Table 2). This significant Pb contamination reveals a non-point nature of the contamination. Therefore, the data do not rule out public health risk due to Pb as residual effect of extensive ambient atmospheric Pb pollution in the country’s recent history. A statistical comparison of the Pb data across the five vegetable types reveals both of the upper quartile and median Pb concentrations to be higher than the Pb standards for all five vegetable types.

**Table 5:** Statistics of the data on Pb

Vegetables	Total count of samples analyzed	Contamination found in samples			Samples with safe levels			Samples yielding ND			Median, µg/g dry weight basis	Upper Quartile, µg/g dry weight basis	Dry weight basis standard for Pb, µg/g
		Total count	% of total count of analyzed samples	Maximum level occurred	Total count	% of total count of analyzed samples	Minimum level detected, dry weight basis	Total count	% of total count of analyzed samples	% of total sample count with safe levels			
White Potato	48	16	33.33	40.968 µg/g dry weight basis	32	66.67	ND	32	66.67	100	7.9015	17.208	0.47619
Green Cabbage	49	8	16.33	29.794 µg/g dry weight basis	41	83.67	0.002 µg/g	37	75.51	90.24	5.9505	12.6865	4.28571
Red Spinach	61	22	36.07	38.439 µg/g dry weight basis	39	63.93	0.049 µg/g	35	57.38	89.74	6.799	13.79	3.75
White Radish	63	18	28.57	36.265 µg/g dry weight basis	45	71.43	1.209 µg/g	44	69.84	97.78	9.377	17.604	2
Green Bean	64	20	31.25	48.43 µg/g dry weight basis	44	68.75	ND	44	68.75	100	5.963	23.425	0.95238
Overall	285	84	29.47	48.43 µg/g dry weight basis	201	70.53	0.002 µg/g	192	67.37	95.52	7.601	15.237	

*ND* not detected (at ppb level for 0.2 g dry sample digested into 100 ml volume)

A cross-comparison between Pb and Cd levels for the five vegetable types in Table 6 reveals similar counts of contaminated White Potato and Green Bean samples. In the remainder three vegetable types the counts of Pb contaminated samples are found higher than those with Cd contamination, while the contrast is steep in the cases of Red Spinach and White Radish. Due to only two samples found contaminated with Cr from the group of 290 samples, this insignificant Cr contamination data are not compared in Table 6.

**Table 6:** Cross-comparison of vegetable types versus *heavy metals* in terms of contamination

*Heavy metal*	% count of contaminated samples among the total count of samples analyzed for each of the *heavy metals*				
	White Potato	Green Cabbage	Red Spinach	White Radish	Green Bean
Pb	33.33 % (out of 48 samples)	16.33 % (out of 49 samples)	36.07 % (out of 61 samples)	28.57 % (out of 63 samples)	31.25 % (out of 64 samples)
Cd	30.61 % (out of 49 samples)	8.16 % (out of 49 samples)	8.33 % (out of 60 samples)	7.81 % (out of 64 samples)	34.37 % (out of 64 samples)

Table 7 summarizes the contamination features for the three *heavy metals* into five geographic regions of the country (the North-West, the North-East, the central, the South-West, and the South-East regions), thus, also constructing the frame for revealing the regional differences in the data. Out of the entire country covered in the sampling (all 64 Districts), only 7 Districts did not produce any contaminated sample. These are Lalmonirhat from the North-West region, Madaripur and Narayanganj from the central region, Bagerhat from the South–West region; and Bandarban, Khagrachari and Noakhali from the South–East region. From among these Districts, Bandarban and Khagrachari form bulk of the country’s Hill Tracts, representing the closest to pristine environments in the country while most of the country’s land-mass being alluvial lowland. Pb contamination was found in samples collected from as many as 49 Districts out of the 64 Districts, which is seconded by Cd (35 out of 64 Districts); while samples with Cr contamination represent the least count of Districts (only two Districts). Therefore, Cr is found to be the least concern compared to Pb and Cd in any of the five demarcated regions, while Pb contamination revealing the biggest concern in four out of the five regions. However, the Pb and Cd contamination in the samples are proportionally existent in all five regions, together with a stark presence of Pb contamination across these regions (such as 20 Districts out of 22 in the central region, or 5 out of 6 Districts in the North–East, and 7 out of 10 Districts in the South–East region).

**Table 7:** Contamination summary revealing regional differences

	*Key features*
*North*-*West region* *17 Districts* Bogra, Chapai Nawabganj, Dinajpur, Gaibandha, Jamalpur, Joypurhat, Kurigram, Lalmonirhat*, Naogaon, Natore, Nilphamari, Panchagarh, Rajshahi, Rangpur, Sherpur, Sirajganj, Thakurgaon	(i) *Pb contamination:* -found in 18 samples -distributed over 12 Districts (averaged 1.5 samples per district) -includes 4 potato, 4 cabbage, 2 spinach, 6 radish, and 2 bean samples *(ii) Cr contamination:* -No samples contaminated *(iii) Cd contamination:* -found in 19 samples -distributed over 13 Districts (averaged 1.46 samples per district) -includes 8 potato, 1 cabbage, 2 spinach, 3 radish, and 5 bean samples
*North*-*East region* *6 Districts* Habiganj, Moulvibazar, Mymensingh, Netrokona, Sunamganj, Sylhet	*(i) Pb contamination:* -found in 8 samples -distributed over 5 Districts (averaged 1.6 samples per district) -includes 3 potato, 2 cabbage, no spinach, 2 radish, and 1 bean samples *(ii) Cr contamination:* -No samples contaminated *(iii) Cd contamination:* -found in 7 samples - distributed over 3 Districts (averaged 2.3 samples per district) -includes 2 potato, 2 cabbage, no spinach, no radish, and 3 bean samples
*Central region* *22 Districts* Brahmanbaria, Chuadanga, Dhaka, Faridpur, Gazipur, Gopalganj, Jessore, Jhenaidah, Kishoreganj, Kushtia, Madaripur*, Magura, Manikganj, Meherpur, Munshiganj, Narail, Narayanganj*, Narshingdi, Pabna, Rajbari, Shariatpur, Tangail	*(i) Pb contamination:* -found in 37 samples -distributed over 20 Districts (averaged 1.85 samples per district) -includes 5 potato, 2 cabbage, 12 spinach, 7 radish, and 11 bean samples *(ii) Cr contamination:* -found in 2 samples -distributed over 2 Districts (averaged 1 sample per district) - these are potato samples *(iii) Cd contamination:* -found in 13 samples - distributed over 10 Districts (averaged 1.3 samples per district) -includes 2 potato, no cabbage, 2 spinach, 1 radish, and 8 bean samples
*South*-*West region* *9 Districts* Bagerhat*, Barguna, Barisal, Bhola, Jhalokathi, Khulna, Patuakhali, Perojpur, Satkhira	*(i) Pb contamination:* -found in 8 samples -distributed over 5 Districts (averaged 1.6 samples per district) -includes 1 potato, no cabbage, 4 spinach, 1 radish, and 2 bean samples *(ii) Cr contamination:* -No samples contaminated *(iii) Cd contamination:* -found in 6 samples -distributed over 4 Districts (averaged 1.5 samples per district) -includes 1 potato, no cabbage, 1 spinach, 1 radish, and 3 bean samples
*South*-*East region* *10 Districts* Bandarban*, Chandpur, Chittagong, Comilla, Cox’s Bazar, Feni, Khagrachari*, Laxmipur, Noakhali*, Rangamati	*(i) Pb contamination:* -found in 13 samples -distributed over 7 Districts (averaged 1.86 samples per district) -includes 3 potato, no cabbage, 4 spinach, 2 radish, and 4 bean samples *(ii) Cr contamination:* -No samples contaminated *(iii) Cd contamination:* -found in 6 samples -distributed over 5 Districts (averaged 1.2 samples per district) -includes 2 potato, 1 cabbage, no spinach, no radish, and 3 bean samples

* Districts that did not contain any sample with contamination for at least one of the *heavy metals*

Taken the total count of the contaminated samples and their Districts of origin for any given *heavy metal* (in Table 7), the statistics on the count of contaminated samples per district of origin reveal a ratio range of 2.3 to 1.00 contaminated samples per district, averaged at 1.56 contaminated samples per district of origin (SD 0.354). This indicates contamination not being a singular event, given the great diversity of vegetable types existing in the sample base with only one sample per vegetable type per district. This statistic of averaged 1.56 contaminated samples per district of origin (SD 0.354) discards any regional significance in the contamination scenario, also due to the wide-spread existence of samples with contamination; while 57 out of the 64 Districts producing at least one sample with contamination. The counts of the contaminated samples across the vegetable types per *heavy metal* per region portray a random scenario with no preference to a particular vegetable type (Table 7).

## Conclusions

The semi-quantitative assessment of Cr contamination in common vegetables in Bangladesh does not substantiate evidence of potential public health risk due to Cr yet, given the particular route of extensive use of tannery-solid-waste-converted protein-concentrates that are highly Cr-contaminated and used in poultry feeds, fish feeds and agricultural application as fertilizer. Given the continuation of this Cr contamination route, it might also be the case that the contamination has not yet been incorporated into the biogeochemical cycle. However, this route of Cr contamination must be arrested completely within as short span as possible in order not to keep potential risk of contaminating the biogeochemical cycle in the future.

The potential point-source nature of exhibited Cd contamination of vegetables (in 17.83 % of the total 286 samples analysed) informs about the scope of managing any existing contamination source by directing efforts to the potential point-sources. However, remediation measures regarding Cd contamination warrants urgent attention given both of the detected upper quartile and median Cd concentrations being higher than the Cd standards in White Potato and Green Bean samples. Nonetheless, Cd contamination of the vegetables is not potentially a wide-spread public health threat if the existing point-sources are traced and ameliorated.

However, the situation regarding Pb is alarming. Although the vast majority of 70.53 % of the analyzed samples reveal safe Pb levels, however, contamination existing in 29.47 % of the samples is significant, together with the nature of the contamination likely being non-point origin. This informs on potential public health risk due to Pb as residual effect of extensive ambient atmospheric Pb pollution in the country’s recent history. Therefore, the contamination scenario regarding Pb demands reinforced efforts on identification, quantification and remediation measures on environmental Pb contamination in Bangladesh.

## Additional file

**Additional file 1: Table S1.** Compilation of detailed data on samples, sample source, sample analysis results, comparison with standards, and comments on the *heavy metals’* status.
