# Supplementary material for: Environmental surveillance of commonly-grown vegetables for investigating potential lead and chromium contamination intensification in Bangladesh
Source: Springerplus. 2016 Oct 18;5(1):1803. doi: 10.1186/s40064-016-3458-9 (PMC5069271; doi:10.1186/s40064-016-3458-9)
Supplement: Supplementary file 1 — Additional file 1: Table S1. Compilation of detailed data on samples, sample source, sample analysis results, comparison with standards, and comments on the heavy metals’ status. [file 40064_2016_3458_MOESM1_ESM.docx]

**Journal:** SpringerPlus

**Article Title:** Environmental surveillance of commonly-grown vegetables for investigating potential Lead and Chromium contamination intensification in Bangladesh

**Authors:** A. M. M. Maruf Hossain^a^*, M. Shahidul Islam^b^, M. Mustafa Mamun^a^, H. M. Al-Jonaed^a^, M. Imran^a^, M. Hasibur Rahman^a^, M. Azizul Islam Kazi^b^, Syed Fazle Elahi^a^

^a^Department of Soil, Water and Environment, Faculty of Biological Sciences, University of Dhaka, Dhaka-1000, Bangladesh;

^b^Analytical Research Division, BCSIR Laboratories, Dr. Qudrat-i-Khuda Road, Dhanmondi, Dhaka-1205, Bangladesh

*Corresponding author email: mueed_abd@yahoo.com [present address: School of Global, Urban and Social Studies, RMIT University, Melbourne VIC 3000, Australia]

**Supplementary Material (Table SM1)**

**Table SM1:** Compilation of detailed data on samples, sample source, sample analysis results, comparison with standards, and comments on the *heavy metals’* status

| **District alphabetical ID-name** | | **Sample serial** | | **Sample source** | **Heavy metals concentrations and comment** | | | | | | | | |
| --- | --- | --- | --- | --- | --- | --- | --- | --- | --- | --- | --- | --- | --- |
| **District serial** | **District name** | **Consecutive serial** | **Vegetable serial** | **Thana name** | **Pb µg/g DW**** | **DW µg/g standard** | **Pb Status** | **Cr µg/g DW** | **µg Cr per 50g FW***** | **Cr Status** | **Cd µg/g DW** | **DW µg/g standard** | **Cd Status** |
| 1 | Barguna | 1 | 3 | Patharghata | 6.998 | 3.75 | Contaminated | ND* | Undetermined | Safe | ND* | 2.5 | Safe |
|  |  | 2 | 4 |  | 10.616 | 2 | Contaminated | ND | Undetermined | Safe | 0.427 | 2 | Safe |
|  |  | 3 | 5 |  | - | - | - | ND | Undetermined | Safe | ND | 0.47619 | Safe |
|  |  |  |  |  |  |  |  |  |  |  |  |  |  |
| 2 | Bagerhat | 4 | 3 | Kachua | ND* | 3.75 | Safe | ND | Undetermined | Safe | ND | 2.5 | Safe |
|  |  | 5 | 4 |  | ND | 2 | Safe | ND | Undetermined | Safe | ND | 2 | Safe |
|  |  |  |  |  |  |  |  |  |  |  |  |  |  |
| 3 | Barisal | 6 | 2 | 2 = Uzirpur; 3,5 = Barisal Sadar | ND | 4.28571 | Safe | ND | Undetermined | Safe | ND | 0.71429 | Safe |
|  |  | 7 | 3 |  | ND | 3.75 | Safe | ND | Undetermined | Safe | ND | 2.5 | Safe |
|  |  | 8 | 5 |  | 6.788 | 0.95238 | Contaminated | ND | Undetermined | Safe | ND | 0.47619 | Safe |
|  |  |  |  |  |  |  |  |  |  |  |  |  |  |
| 4 | Bogra | 9 | 1 | 1,4,5 = Sherpur; 2,3 = Shajahanpur | 4.477 | 0.47619 | Contaminated | 1.849 | 19.4145 | Safe | ND | 0.47619 | Safe |
|  |  | 10 | 2 |  | ND | 4.28571 | Safe | ND | Undetermined | Safe | 0.395 | 0.71429 | Safe |
|  |  | 11 | 3 |  | ND | 3.75 | Safe | ND | Undetermined | Safe | 0.763 | 2.5 | Safe |
|  |  | 12 | 4 |  | ND | 2 | Safe | ND | Undetermined | Safe | ND | 2 | Safe |
|  |  | 13 | 5 |  | ND | 0.95238 | Safe | ND | Undetermined | Safe | 0.645 | 0.47619 | Contaminated |
|  |  |  |  |  |  |  |  |  |  |  |  |  |  |
| 5 | Bandarban | 14 | 3 | Banderban sadar | 3.31 | 3.75 | Safe | ND | Undetermined | Safe | 0.445 | 2.5 | Safe |
|  |  | 15 | 4 |  | ND | 2 | Safe | ND | Undetermined | Safe | ND | 2 | Safe |
|  |  | 16 | 5 |  | ND | 0.95238 | Safe | ND | Undetermined | Safe | - | - | - |
|  |  |  |  |  |  |  |  |  |  |  |  |  |  |
| 6 | Bhola | 17 | 2 | Bhola Sadar | ND | 4.28571 | Safe | ND | Undetermined | Safe | ND | 0.71429 | Safe |
|  |  | 18 | 3 |  | 30.269 | 3.75 | Contaminated | ND | Undetermined | Safe | 0.577 | 2.5 | Safe |
|  |  | 19 | 4 |  | ND | 2 | Safe | ND | Undetermined | Safe | ND | 2 | Safe |
|  |  | 20 | 5 |  | 5.463 | 0.95238 | Contaminated | ND | Undetermined | Safe | ND | 0.47619 | Safe |
|  |  |  |  |  |  |  |  |  |  |  |  |  |  |
| 7 | Brahmanbaria | 21 | 1 | Kasba | 8.26 | 0.47619 | Contaminated | ND | Undetermined | Safe | ND | 0.47619 | Safe |
|  |  | 22 | 2 |  | 14.765 | 4.28571 | Contaminated | ND | Undetermined | Safe | ND | 0.71429 | Safe |
|  |  | 23 | 3 |  | ND | 3.75 | Safe | ND | Undetermined | Safe | ND | 2.5 | Safe |
|  |  | 24 | 4 |  | ND | 2 | Safe | ND | Undetermined | Safe | ND | 2 | Safe |
|  |  | 25 | 5 |  | 1.136 | 0.95238 | Contaminated | 22.585 | 118.57125 | Safe | 0.663 | 0.47619 | Contaminated |
|  |  |  |  |  |  |  |  |  |  |  |  |  |  |
| 8 | Chandpur | 26 | 1 | Faridgonj | ND | 0.47619 | Safe | 5.248 | 55.104 | Safe | ND | 0.47619 | Safe |
|  |  | 27 | 3 |  | ND | 3.75 | Safe | ND | Undetermined | Safe | 0.15 | 2.5 | Safe |
|  |  | 28 | 4 |  | ND | 2 | Safe | ND | Undetermined | Safe | ND | 2 | Safe |
|  |  | 29 | 5 |  | 4.014 | 0.95238 | Contaminated | 1.09 | 5.7225 | Safe | 0.991 | 0.47619 | Contaminated |
|  |  |  |  |  |  |  |  |  |  |  |  |  |  |
| 9 | Chapainawabganj | 30 | 2 | Chapai sadar | ND | 4.28571 | Safe | ND | Undetermined | Safe | 0.297 | 0.71429 | Safe |
|  |  | 31 | 3 |  | 4.341 | 3.75 | Contaminated | ND | Undetermined | Safe | 0.15 | 2.5 | Safe |
|  |  | 32 | 4 |  | ND | 2 | Safe | ND | Undetermined | Safe | 0.68 | 2 | Safe |
|  |  | 33 | 5 |  | ND | 0.95238 | Safe | ND | Undetermined | Safe | 0.672 | 0.47619 | Contaminated |
|  |  |  |  |  |  |  |  |  |  |  |  |  |  |
| 10 | Cox's Bazar | 34 | 1 | Cox's Bazar | - | - | - | ND | Undetermined | Safe | 0.049 | 0.47619 | Safe |
|  |  | 35 | 3 |  | 11.176 | 3.75 | Contaminated | ND | Undetermined | Safe | ND | 2.5 | Safe |
|  |  | 36 | 4 |  | 3.35 | 2 | Contaminated | ND | Undetermined | Safe | ND | 2 | Safe |
|  |  |  |  |  |  |  |  |  |  |  |  |  |  |
| 11 | Chittagong | 37 | 1 | Shitakunda | 18.182 | 0.47619 | Contaminated | ND | Undetermined | Safe | ND | 0.47619 | Safe |
|  |  | 38 | 2 |  | 0.002 | 4.28571 | Safe | ND | Undetermined | Safe | ND | 0.71429 | Safe |
|  |  | 39 | 3 |  | 38.439 | 3.75 | Contaminated | ND | Undetermined | Safe | ND | 2.5 | Safe |
|  |  | 40 | 4 |  | 36.265 | 2 | Contaminated | ND | Undetermined | Safe | 0.541 | 2 | Safe |
|  |  | 41 | 5 |  | 48.43 | 0.95238 | Contaminated | ND | Undetermined | Safe | 0.638 | 0.47619 | Contaminated |
|  |  |  |  |  |  |  |  |  |  |  |  |  |  |
| 12 | Comilla | 42 | 2 | Muradnagar | ND | 4.28571 | Safe | ND | Undetermined | Safe | ND | 0.71429 | Safe |
|  |  | 43 | 3 |  | 5.195 | 3.75 | Contaminated | ND | Undetermined | Safe | ND | 2.5 | Safe |
|  |  | 44 | 4 |  | ND | 2 | Safe | ND | Undetermined | Safe | 0.149 | 2 | Safe |
|  |  | 45 | 5 |  | ND | 0.95238 | Safe | ND | Undetermined | Safe | ND | 0.47619 | Safe |
|  |  |  |  |  |  |  |  |  |  |  |  |  |  |
| 13 | Chuadanga | 46 | 1 | Alamdanga | ND | 0.47619 | Safe | ND | Undetermined | Safe | 1.064 | 0.47619 | Contaminated |
|  |  | 47 | 2 |  | ND | 4.28571 | Safe | ND | Undetermined | Safe | ND | 0.71429 | Safe |
|  |  | 48 | 3 |  | ND | 3.75 | Safe | ND | Undetermined | Safe | 0.345 | 2.5 | Safe |
|  |  | 49 | 4 |  | ND | 2 | Safe | ND | Undetermined | Safe | 0.245 | 2 | Safe |
|  |  | 50 | 5 |  | ND | 0.95238 | Safe | ND | Undetermined | Safe | 1.137 | 0.47619 | Contaminated |
|  |  |  |  |  |  |  |  |  |  |  |  |  |  |
| 14-1 | Dhaka 1 | 51 | 1 | Ashulia | ND | 0.47619 | Safe | 1.377 | 14.4585 | Safe | ND | 0.47619 | Safe |
|  |  | 52 | 3 |  | 6.182 | 3.75 | Contaminated | ND | Undetermined | Safe | ND | 2.5 | Safe |
|  |  | 53 | 4 |  | 8.683 | 2 | Contaminated | ND | Undetermined | Safe | ND | 2 | Safe |
|  |  | 54 | 5 |  | ND | 0.95238 | Safe | 1.39 | 7.2975 | Safe | ND | 0.47619 | Safe |
|  |  |  |  |  |  |  |  |  |  |  |  |  |  |
| 14-2 | Dhaka 2 | 55 | 3 | Keranigonj | ND | 3.75 | Safe | ND | Undetermined | Safe | ND | 2.5 | Safe |
|  |  | 56 | 4 |  | ND | 2 | Safe | ND | Undetermined | Safe | ND | 2 | Safe |
|  |  | 57 | 5 |  | ND | 0.95238 | Safe | ND | Undetermined | Safe | ND | 0.47619 | Safe |
|  |  |  |  |  |  |  |  |  |  |  |  |  |  |
| 15 | Dinajpur | 58 | 1 | Birgonj | ND | 0.47619 | Safe | ND | Undetermined | Safe | ND | 0.47619 | Safe |
|  |  | 59 | 2 |  | 10.918 | 4.28571 | Contaminated | ND | Undetermined | Safe | ND | 0.71429 | Safe |
|  |  | 60 | 3 |  | ND | 3.75 | Safe | ND | Undetermined | Safe | 0.196 | 2.5 | Safe |
|  |  | 61 | 4 |  | 12.173 | 2 | Contaminated | ND | Undetermined | Safe | 0.252 | 2 | Safe |
|  |  | 62 | 5 |  | ND | 0.95238 | Safe | ND | Undetermined | Safe | ND | 0.47619 | Safe |
|  |  |  |  |  |  |  |  |  |  |  |  |  |  |
| 16 | Faridpur | 63 | 1 | Alfadanga | ND | 0.47619 | Safe | ND | Undetermined | Safe | ND | 0.47619 | Safe |
|  |  | 64 | 2 |  | ND | 4.28571 | Safe | ND | Undetermined | Safe | ND | 0.71429 | Safe |
|  |  | 65 | 3 |  | 5.853 | 3.75 | Contaminated | ND | Undetermined | Safe | ND | 2.5 | Safe |
|  |  | 66 | 4 |  | 3.85 | 2 | Contaminated | ND | Undetermined | Safe | ND | 2 | Safe |
|  |  | 67 | 5 |  | 23.617 | 0.95238 | Contaminated | ND | Undetermined | Safe | 2.143 | 0.47619 | Contaminated |
|  |  |  |  |  |  |  |  |  |  |  |  |  |  |
| 17 | Feni | 68 | 1 | Dagonbhuiyaan | 37.362 | 0.47619 | Contaminated | ND | Undetermined | Safe | 1.107 | 0.47619 | Contaminated |
|  |  | 69 | 3 |  | 12.415 | 3.75 | Contaminated | ND | Undetermined | Safe | ND | 2.5 | Safe |
|  |  | 70 | 4 |  | - | - | - | ND | Undetermined | Safe | 1.067 | 2 | Safe |
|  |  | 71 | 5 |  | 4.938 | 0.95238 | Contaminated | 1.246 | 6.5415 | Safe | ND | 0.47619 | Safe |
|  |  |  |  |  |  |  |  |  |  |  |  |  |  |
| 18 | Gaibandha | 72 | 1 | Gobindogonj | ND | 0.47619 | Safe | ND | Undetermined | Safe | 0.426 | 0.47619 | Safe |
|  |  | 73 | 2 |  | 13.111 | 4.28571 | Contaminated | ND | Undetermined | Safe | ND | 0.71429 | Safe |
|  |  | 74 | 3 |  | ND | 3.75 | Safe | ND | Undetermined | Safe | ND | 2.5 | Safe |
|  |  | 75 | 4 |  | ND | 2 | Safe | ND | Undetermined | Safe | ND | 2 | Safe |
|  |  | 76 | 5 |  | 1.487 | 0.95238 | Contaminated | 0.144 | 0.756 | Safe | 1.103 | 0.47619 | Contaminated |
|  |  |  |  |  |  |  |  |  |  |  |  |  |  |
| 19 | Gazipur | 77 | 2 | Kapashia | ND | 4.28571 | Safe | ND | Undetermined | Safe | ND | 0.71429 | Safe |
|  |  | 78 | 3 |  | 20.467 | 3.75 | Contaminated | ND | Undetermined | Safe | 1.192 | 2.5 | Safe |
|  |  | 79 | 4 |  | ND | 2 | Safe | ND | Undetermined | Safe | 0.435 | 2 | Safe |
|  |  | 80 | 5 |  | 21.034 | 0.95238 | Contaminated | ND | Undetermined | Safe | ND | 0.47619 | Safe |
|  |  |  |  |  |  |  |  |  |  |  |  |  |  |
| 20 | Gopalganj | 81 | 1 | Kashiani | ND | 0.47619 | Safe | 29.142 | 305.991 | Contaminated | ND | 0.47619 | Safe |
|  |  | 82 | 2 |  | ND | 4.28571 | Safe | 1.915 | 6.7025 | Safe | ND | 0.71429 | Safe |
|  |  | 83 | 3 |  | ND | 3.75 | Safe | ND | Undetermined | Safe | ND | 2.5 | Safe |
|  |  | 84 | 4 |  | ND | 2 | Safe | ND | Undetermined | Safe | ND | 2 | Safe |
|  |  | 85 | 5 |  | 30.78 | 0.95238 | Contaminated | 1.854 | 9.7335 | Safe | 0.341 | 0.47619 | Safe |
|  |  |  |  |  |  |  |  |  |  |  |  |  |  |
| 21 | Habiganj | 86 | 1 | Habiganj | 19.99 | 0.47619 | Contaminated | 0.945 | 9.9225 | Safe | 3.58 | 0.47619 | Contaminated |
|  |  | 87 | 2 |  | 29.794 | 4.28571 | Contaminated | ND | Undetermined | Safe | 0.737 | 0.71429 | Contaminated |
|  |  | 88 | 3 |  | ND | 3.75 | Safe | ND | Undetermined | Safe | 0.609 | 2.5 | Safe |
|  |  | 89 | 4 |  | ND | 2 | Safe | 7.711 | 7.711 | Safe | ND | 2 | Safe |
|  |  | 90 | 5 |  | ND | 0.95238 | Safe | ND | Undetermined | Safe | 0.576 | 0.47619 | Contaminated |
|  |  |  |  |  |  |  |  |  |  |  |  |  |  |
| 22 | Jessore | 91 | 1 | Chowgacha | 40.968 | 0.47619 | Contaminated | ND | Undetermined | Safe | ND | 0.47619 | Safe |
|  |  | 92 | 4 |  | 13.882 | 2 | Contaminated | ND | Undetermined | Safe | ND | 2 | Safe |
|  |  | 93 | 5 |  | ND | 0.95238 | Safe | ND | Undetermined | Safe | 0.243 | 0.47619 | Safe |
|  |  |  |  |  |  |  |  |  |  |  |  |  |  |
| 23 | Jhalokathi | 94 | 1 | Nalchity | 7.456 | 0.47619 | Contaminated | 0.566 | 5.943 | Safe | ND | 0.47619 | Safe |
|  |  | 95 | 2 |  | ND | 4.28571 | Safe | ND | Undetermined | Safe | ND | 0.71429 | Safe |
|  |  | 96 | 3 |  | 13.757 | 3.75 | Contaminated | ND | Undetermined | Safe | ND | 2.5 | Safe |
|  |  | 97 | 4 |  | ND | 2 | Safe | ND | Undetermined | Safe | ND | 2 | Safe |
|  |  | 98 | 5 |  | ND | 0.95238 | Safe | ND | Undetermined | Safe | ND | 0.47619 | Safe |
|  |  |  |  |  |  |  |  |  |  |  |  |  |  |
| 24 | Jamalpur | 99 | 1 | Jamalpur | ND | 0.47619 | Safe | 2.399 | 25.1895 | Safe | 0.96 | 0.47619 | Contaminated |
|  |  | 100 | 3 |  | ND | 3.75 | Safe | ND | Undetermined | Safe | ND | 2.5 | Safe |
|  |  | 101 | 4 |  | 21.972 | 2 | Contaminated | ND | Undetermined | Safe | 3.638 | 2 | Contaminated |
|  |  | 102 | 5 |  | 3.223 | 0.95238 | Contaminated | 24.938 | 130.9245 | Safe | 1.091 | 0.47619 | Contaminated |
|  |  |  |  |  |  |  |  |  |  |  |  |  |  |
| 25 | Joypurhat | 103 | 1 | Joypurhat | 8.576 | 0.47619 | Contaminated | ND | Undetermined | Safe | 0.493 | 0.47619 | Contaminated |
|  |  | 104 | 2 |  | ND | 4.28571 | Safe | ND | Undetermined | Safe | ND | 0.71429 | Safe |
|  |  | 105 | 3 |  | ND | 3.75 | Safe | ND | Undetermined | Safe | ND | 2.5 | Safe |
|  |  | 106 | 4 |  | 4.425 | 2 | Contaminated | ND | Undetermined | Safe | ND | 2 | Safe |
|  |  | 107 | 5 |  | ND | 0.95238 | Safe | ND | Undetermined | Safe | ND | 0.47619 | Safe |
|  |  |  |  |  |  |  |  |  |  |  |  |  |  |
| 26 | Jhenaidah | 108 | 1 | Jhenaidah | ND | 0.47619 | Safe | 8.835 | 92.7675 | Safe | 0.243 | 0.47619 | Safe |
|  |  | 109 | 2 |  | 4.726 | 4.28571 | Contaminated | ND | Undetermined | Safe | 0.448 | 0.71429 | Safe |
|  |  | 110 | 3 |  | 28.888 | 3.75 | Contaminated | ND | Undetermined | Safe | 0.098 | 2.5 | Safe |
|  |  | 111 | 4 |  | 23.98 | 2 | Contaminated | ND | Undetermined | Safe | 0.746 | 2 | Safe |
|  |  | 112 | 5 |  | - | - | - | 1.388 | 7.287 | Safe | 0.297 | 0.47619 | Safe |
|  |  |  |  |  |  |  |  |  |  |  |  |  |  |
| 27 | Khulna | 113 | 5 | Rupsha | ND | 0.95238 | Safe | ND | Undetermined | Safe | 0.945 | 0.47619 | Contaminated |
|  |  |  |  |  |  |  |  |  |  |  |  |  |  |
| 28 | Kurigram | 114 | 1 | Bhurungamari | ND | 0.47619 | Safe | ND | Undetermined | Safe | 0.2 | 0.47619 | Safe |
|  |  | 115 | 2 |  | 11.413 | 4.28571 | Contaminated | ND | Undetermined | Safe | 0.543 | 0.71429 | Safe |
|  |  | 116 | 3 |  | ND | 3.75 | Safe | ND | Undetermined | Safe | 1.592 | 2.5 | Safe |
|  |  | 117 | 4 |  | ND | 2 | Safe | ND | Undetermined | Safe | ND | 2 | Safe |
|  |  | 118 | 5 |  | ND | 0.95238 | Safe | ND | Undetermined | Safe | 0.149 | 0.47619 | Safe |
|  |  |  |  |  |  |  |  |  |  |  |  |  |  |
| 29 | Khagrachari | 119 | 1 | Khagrachari | ND | 0.47619 | Safe | ND | Undetermined | Safe | ND | 0.47619 | Safe |
|  |  | 120 | 2 |  | ND | 4.28571 | Safe | ND | Undetermined | Safe | 0.099 | 0.71429 | Safe |
|  |  | 121 | 3 |  | ND | 3.75 | Safe | ND | Undetermined | Safe | ND | 2.5 | Safe |
|  |  | 122 | 4 |  | ND | 2 | Safe | ND | Undetermined | Safe | 1.52 | 2 | Safe |
|  |  | 123 | 5 |  | ND | 0.95238 | Safe | ND | Undetermined | Safe | ND | 0.47619 | Safe |
|  |  |  |  |  |  |  |  |  |  |  |  |  |  |
| 30 | Kushtia | 124 | 1 | Kushtia sadar | 5.314 | 0.47619 | Contaminated | ND | Undetermined | Safe | ND | 0.47619 | Safe |
|  |  | 125 | 2 |  | 3.96 | 4.28571 | Safe | 7.426 | 25.991 | Safe | ND | 0.71429 | Safe |
|  |  | 126 | 3 |  | 3.9 | 3.75 | Contaminated | ND | Undetermined | Safe | 0.6 | 2.5 | Safe |
|  |  | 127 | 4 |  | 3.953 | 2 | Contaminated | ND | Undetermined | Safe | 3.063 | 2 | Contaminated |
|  |  | 128 | 5 |  | 2.177 | 0.95238 | Contaminated | ND | Undetermined | Safe | 1.158 | 0.47619 | Contaminated |
|  |  |  |  |  |  |  |  |  |  |  |  |  |  |
| 31-1 | Kishoreganj | 129 | 1 | Kotiyadi | ND | 0.47619 | Safe | ND | Undetermined | Safe | 0.35 | 0.47619 | Safe |
|  |  | 130 | 2 |  | ND | 4.28571 | Safe | 2.21 | 7.735 | Safe | ND | 0.71429 | Safe |
|  |  | 131 | 4 |  | ND | 2 | Safe | ND | Undetermined | Safe | ND | 2 | Safe |
|  |  | 132 | 5 |  | ND | 0.95238 | Safe | ND | Undetermined | Safe | ND | 0.47619 | Safe |
|  |  |  |  |  |  |  |  |  |  |  |  |  |  |
| 31-2 | Kishoreganj | 133 | 1 | Itna | ND | 0.47619 | Safe | 3.98 | 41.79 | Safe | ND | 0.47619 | Safe |
|  |  | 134 | 2 |  | ND | 4.28571 | Safe | 0.25 | 0.875 | Safe | ND | 0.71429 | Safe |
|  |  | 135 | 3 |  | 8.858 | 3.75 | Contaminated | ND | Undetermined | Safe | 3.74 | 2.5 | Contaminated |
|  |  | 136 | 4 |  | ND | 2 | Safe | 0.303 | 0.303 | Safe | 1.517 | 2 | Safe |
|  |  | 137 | 5 |  | ND | 0.95238 | Safe | ND | Undetermined | Safe | ND | 0.47619 | Safe |
|  |  |  |  |  |  |  |  |  |  |  |  |  |  |
| 32 | Lakshmipur | 138 | 3 | Lakshmipur | ND | 3.75 | Safe | ND | Undetermined | Safe | 1.051 | 2.5 | Safe |
|  |  | 139 | 4 |  | ND | 2 | Safe | ND | Undetermined | Safe | 0.573 | 2 | Safe |
|  |  | 140 | 5 |  | 1.141 | 0.95238 | Contaminated | ND | Undetermined | Safe | 0.942 | 0.47619 | Contaminated |
|  |  |  |  |  |  |  |  |  |  |  |  |  |  |
| 33 | Lalmonirhat | 141 | 1 | Kaligonj | ND | 0.47619 | Safe | 8.004 | 84.042 | Safe | ND | 0.47619 | Safe |
|  |  | 142 | 2 |  | ND | 4.28571 | Safe | ND | Undetermined | Safe | 0.098 | 0.71429 | Safe |
|  |  | 143 | 3 |  | ND | 3.75 | Safe | ND | Undetermined | Safe | ND | 2.5 | Safe |
|  |  | 144 | 4 |  | ND | 2 | Safe | ND | Undetermined | Safe | 0.659 | 2 | Safe |
|  |  | 145 | 5 |  | ND | 0.95238 | Safe | 0.391 | 2.05275 | Safe | 0.098 | 0.47619 | Safe |
|  |  |  |  |  |  |  |  |  |  |  |  |  |  |
| 34 | Madaripur | 146 | 1 | Madaripur | - | - | - | ND | Undetermined | Safe | ND | 0.47619 | Safe |
|  |  | 147 | 3 |  | ND | 3.75 | Safe | 0.288 | 1.152 | Safe | ND | 2.5 | Safe |
|  |  | 148 | 4 |  | ND | 2 | Safe | 1.808 | 1.808 | Safe | 1.142 | 2 | Safe |
|  |  | 149 | 5 |  | ND | 0.95238 | Safe | 0.001 | 0.00525 | Safe | ND | 0.47619 | Safe |
|  |  |  |  |  |  |  |  |  |  |  |  |  |  |
| 35 | Magura | 150 | 1 | Shalikha | ND | 0.47619 | Safe | ND | Undetermined | Safe | ND | 0.47619 | Safe |
|  |  | 151 | 2 |  | ND | 4.28571 | Safe | ND | Undetermined | Safe | 0.151 | 0.71429 | Safe |
|  |  | 152 | 3 |  | ND | 3.75 | Safe | ND | Undetermined | Safe | 0.15 | 2.5 | Safe |
|  |  | 153 | 4 |  | ND | 2 | Safe | ND | Undetermined | Safe | ND | 2 | Safe |
|  |  | 154 | 5 |  | 22.849 | 0.95238 | Contaminated | ND | Undetermined | Safe | 4.111 | 0.47619 | Contaminated |
|  |  |  |  |  |  |  |  |  |  |  |  |  |  |
| 36 | Meherpur | 155 | 1 | Meherpur | ND | 0.47619 | Safe | ND | Undetermined | Safe | ND | 0.47619 | Safe |
|  |  | 156 | 2 |  | ND | 4.28571 | Safe | 0.802 | 2.807 | Safe | ND | 0.71429 | Safe |
|  |  | 157 | 4 |  | ND | 2 | Safe | ND | Undetermined | Safe | ND | 2 | Safe |
|  |  | 158 | 5 |  | 15.459 | 0.95238 | Contaminated | ND | Undetermined | Safe | 4.133 | 0.47619 | Contaminated |
|  |  |  |  |  |  |  |  |  |  |  |  |  |  |
| 37 | Moulvibazar | 159 | 1 | Srimangal | ND | 0.47619 | Safe | 2.775 | 29.1375 | Safe | ND | 0.47619 | Safe |
|  |  | 160 | 3 |  | ND | 3.75 | Safe | ND | Undetermined | Safe | 0.05 | 2.5 | Safe |
|  |  | 161 | 4 |  | 9.377 | 2 | Contaminated | ND | Undetermined | Safe | 0.349 | 2 | Safe |
|  |  | 162 | 5 |  | ND | 0.95238 | Safe | ND | Undetermined | Safe | ND | 0.47619 | Safe |
|  |  |  |  |  |  |  |  |  |  |  |  |  |  |
| 38 | Mymensingh | 163 | 1 | Phulpur | ND | 0.47619 | Safe | ND | Undetermined | Safe | 0.701 | 0.47619 | Contaminated |
|  |  | 164 | 2 |  | ND | 4.28571 | Safe | ND | Undetermined | Safe | 0.799 | 0.71429 | Contaminated |
|  |  | 165 | 3 |  | 3.482 | 3.75 | Safe | ND | Undetermined | Safe | 0.687 | 2.5 | Safe |
|  |  | 166 | 4 |  | ND | 2 | Safe | ND | Undetermined | Safe | ND | 2 | Safe |
|  |  | 167 | 5 |  | ND | 0.95238 | Safe | ND | Undetermined | Safe | 0.903 | 0.47619 | Contaminated |
|  |  |  |  |  |  |  |  |  |  |  |  |  |  |
| 39 | Manikganj | 168 | 1 | Manikgonj | ND | 0.47619 | Safe | ND | Undetermined | Safe | ND | 0.47619 | Safe |
|  |  | 169 | 2 |  | ND | 4.28571 | Safe | ND | Undetermined | Safe | ND | 0.71429 | Safe |
|  |  | 170 | 3 |  | ND | 3.75 | Safe | ND | Undetermined | Safe | ND | 2.5 | Safe |
|  |  | 171 | 4 |  | ND | 2 | Safe | ND | Undetermined | Safe | 0.2 | 2 | Safe |
|  |  | 172 | 5 |  | 28.258 | 0.95238 | Contaminated | ND | Undetermined | Safe | 5.092 | 0.47619 | Contaminated |
|  |  |  |  |  |  |  |  |  |  |  |  |  |  |
| 40 | Munshiganj | 173 | 1 | Munshigonj | ND | 0.47619 | Safe | 21.155 | 222.1275 | Contaminated | ND | 0.47619 | Safe |
|  |  | 174 | 2 |  | ND | 4.28571 | Safe | ND | Undetermined | Safe | ND | 0.71429 | Safe |
|  |  | 175 | 3 |  | 4.144 | 3.75 | Contaminated | ND | Undetermined | Safe | ND | 2.5 | Safe |
|  |  | 176 | 4 |  | 8.965 | 2 | Contaminated | ND | Undetermined | Safe | 0.668 | 2 | Safe |
|  |  | 177 | 5 |  | ND | 0.95238 | Safe | ND | Undetermined | Safe | ND | 0.47619 | Safe |
|  |  |  |  |  |  |  |  |  |  |  |  |  |  |
| 41 | Narail | 178 | 2 | 2,5 = Narail; 3,4 = Lohagara | ND | 4.28571 | Safe | 0.952 | 3.332 | Safe | ND | 0.71429 | Safe |
|  |  | 179 | 3 |  | 4.017 | 3.75 | Contaminated | ND | Undetermined | Safe | 1.307 | 2.5 | Safe |
|  |  | 180 | 4 |  | 6.068 | 2 | Contaminated | ND | Undetermined | Safe | ND | 2 | Safe |
|  |  | 181 | 5 |  | ND | 0.95238 | Safe | ND | Undetermined | Safe | ND | 0.47619 | Safe |
|  |  |  |  |  |  |  |  |  |  |  |  |  |  |
| 42 | Narayanganj | 182 | 2 | Sonargaon | ND | 4.28571 | Safe | 0.241 | 0.8435 | Safe | ND | 0.71429 | Safe |
|  |  | 183 | 3 |  | ND | 3.75 | Safe | ND | Undetermined | Safe | 0.05 | 2.5 | Safe |
|  |  | 184 | 4 |  | ND | 2 | Safe | ND | Undetermined | Safe | ND | 2 | Safe |
|  |  | 185 | 5 |  | ND | 0.95238 | Safe | ND | Undetermined | Safe | ND | 0.47619 | Safe |
|  |  |  |  |  |  |  |  |  |  |  |  |  |  |
| 43 | Noakhali | 186 | 3 | Companygonj | ND | 3.75 | Safe | ND | Undetermined | Safe | 0.202 | 2.5 | Safe |
|  |  | 187 | 4 |  | ND | 2 | Safe | ND | Undetermined | Safe | 1.347 | 2 | Safe |
|  |  | 188 | 5 |  | ND | 0.95238 | Safe | ND | Undetermined | Safe | ND | 0.47619 | Safe |
|  |  |  |  |  |  |  |  |  |  |  |  |  |  |
| 44 | Naogaon | 189 | 1 | Manda | ND | 0.47619 | Safe | ND | Undetermined | Safe | ND | 0.47619 | Safe |
|  |  | 190 | 2 |  | ND | 4.28571 | Safe | ND | Undetermined | Safe | ND | 0.71429 | Safe |
|  |  | 191 | 3 |  | ND | 3.75 | Safe | 3.593 | 14.372 | Safe | 4.872 | 2.5 | Contaminated |
|  |  | 192 | 4 |  | ND | 2 | Safe | ND | Undetermined | Safe | ND | 2 | Safe |
|  |  | 193 | 5 |  | ND | 0.95238 | Safe | 1.649 | 8.65725 | Safe | ND | 0.47619 | Safe |
|  |  |  |  |  |  |  |  |  |  |  |  |  |  |
| 45-1 | Narsingdi | 194 | 1 | Raipura | ND | 0.47619 | Safe | ND | Undetermined | Safe | 1.93 | 0.47619 | Contaminated |
|  |  | 195 | 2 |  | ND | 4.28571 | Safe | ND | Undetermined | Safe | 0.152 | 0.71429 | Safe |
|  |  | 196 | 3 |  | ND | 3.75 | Safe | ND | Undetermined | Safe | 1.938 | 2.5 | Safe |
|  |  | 197 | 4 |  | ND | 2 | Safe | ND | Undetermined | Safe | ND | 2 | Safe |
|  |  | 198 | 5 |  | 47.793 | 0.95238 | Contaminated | ND | Undetermined | Safe | 4.746 | 0.47619 | Contaminated |
|  |  |  |  |  |  |  |  |  |  |  |  |  |  |
| 45-2 | Narsingdi | 199 | 1 | 1,4 = Raipura; 2,3,5 = Belabo | ND | 0.47619 | Safe | ND | Undetermined | Safe | ND | 0.47619 | Safe |
|  |  | 200 | 2 |  | ND | 4.28571 | Safe | 1.381 | 4.8335 | Safe | ND | 0.71429 | Safe |
|  |  | 201 | 3 |  | 13.889 | 3.75 | Contaminated | ND | Undetermined | Safe | 3.947 | 2.5 | Contaminated |
|  |  | 202 | 4 |  | ND | 2 | Safe | ND | Undetermined | Safe | ND | 2 | Safe |
|  |  | 203 | 5 |  | ND | 0.95238 | Safe | ND | Undetermined | Safe | ND | 0.47619 | Safe |
|  |  |  |  |  |  |  |  |  |  |  |  |  |  |
| 46 | Natore | 204 | 1 | Lalpur | ND | 0.47619 | Safe | 0.746 | 7.833 | Safe | 1.243 | 0.47619 | Contaminated |
|  |  | 205 | 3 |  | ND | 3.75 | Safe | 0.246 | 0.984 | Safe | 0.885 | 2.5 | Safe |
|  |  | 206 | 4 |  | 8.126 | 2 | Contaminated | ND | Undetermined | Safe | 3.241 | 2 | Contaminated |
|  |  | 207 | 5 |  | ND | 0.95238 | Safe | ND | Undetermined | Safe | ND | 0.47619 | Safe |
|  |  |  |  |  |  |  |  |  |  |  |  |  |  |
| 47 | Netrokona | 208 | 1 | Kendua | 4.015 | 0.47619 | Contaminated | - | - | - | - | - | - |
|  |  | 209 | 4 |  | ND | 2 | Safe | 0.69 | 0.69 | Safe | ND | 2 | Safe |
|  |  | 210 | 5 |  | ND | 0.95238 | Safe | - | - | - | - | - | - |
|  |  |  |  |  |  |  |  |  |  |  |  |  |  |
| 48 | Nilphamari | 211 | 1 | Saidpur | ND | 0.47619 | Safe | ND | Undetermined | Safe | 0.353 | 0.47619 | Safe |
|  |  | 212 | 2 |  | 2.795 | 4.28571 | Safe | ND | Undetermined | Safe | ND | 0.71429 | Safe |
|  |  | 213 | 3 |  | 0.049 | 3.75 | Safe | ND | Undetermined | Safe | ND | 2.5 | Safe |
|  |  | 214 | 4 |  | 17.604 | 2 | Contaminated | ND | Undetermined | Safe | 1.436 | 2 | Safe |
|  |  | 215 | 5 |  | ND | 0.95238 | Safe | ND | Undetermined | Safe | 0.343 | 0.47619 | Safe |
|  |  |  |  |  |  |  |  |  |  |  |  |  |  |
| 49 | Pabna | 216 | 1 | 1,2,5 = Chatmahar; 3,4 = Iswardi | 1.13 | 0.47619 | Contaminated | 1.375 | 14.4375 | Safe | ND | 0.47619 | Safe |
|  |  | 217 | 2 |  | ND | 4.28571 | Safe | ND | Undetermined | Safe | ND | 0.71429 | Safe |
|  |  | 218 | 3 |  | 12.562 | 3.75 | Contaminated | ND | Undetermined | Safe | ND | 2.5 | Safe |
|  |  | 219 | 4 |  | 1.209 | 2 | Safe | ND | Undetermined | Safe | ND | 2 | Safe |
|  |  | 220 | 5 |  | 6.463 | 0.95238 | Contaminated | 0.146 | 0.7665 | Safe | ND | 0.47619 | Safe |
|  |  |  |  |  |  |  |  |  |  |  |  |  |  |
| 50 | Panchagarh | 221 | 1 | Atwari | - | - | - | ND | Undetermined | Safe | 0.428 | 0.47619 | Safe |
|  |  | 222 | 2 |  | ND | 4.28571 | Safe | ND | Undetermined | Safe | ND | 0.71429 | Safe |
|  |  | 223 | 3 |  | ND | 3.75 | Safe | ND | Undetermined | Safe | 0.645 | 2.5 | Safe |
|  |  | 224 | 4 |  | ND | 2 | Safe | ND | Undetermined | Safe | ND | 2 | Safe |
|  |  | 225 | 5 |  | ND | 0.95238 | Safe | ND | Undetermined | Safe | 0.522 | 0.47619 | Contaminated |
|  |  |  |  |  |  |  |  |  |  |  |  |  |  |
| 51 | Potuakhali | 226 | 2 | Dashmina | ND | 4.28571 | Safe | ND | Undetermined | Safe | ND | 0.71429 | Safe |
|  |  | 227 | 3 |  | ND | 3.75 | Safe | ND | Undetermined | Safe | ND | 2.5 | Safe |
|  |  | 228 | 4 |  | ND | 2 | Safe | 25.173 | 25.173 | Safe | ND | 2 | Safe |
|  |  | 229 | 5 |  | ND | 0.95238 | Safe | ND | Undetermined | Safe | 0.851 | 0.47619 | Contaminated |
|  |  |  |  |  |  |  |  |  |  |  |  |  |  |
| 52 | Pirojpur | 230 | 1 | Najirpur | ND | 0.47619 | Safe | 1.637 | 17.1885 | Safe | 0.843 | 0.47619 | Contaminated |
|  |  | 231 | 2 |  | ND | 4.28571 | Safe | ND | Undetermined | Safe | ND | 0.71429 | Safe |
|  |  | 232 | 3 |  | 19.146 | 3.75 | Contaminated | ND | Undetermined | Safe | 4.175 | 2.5 | Contaminated |
|  |  | 233 | 4 |  | - | - | - | 2.979 | 2.979 | Safe | - | - | - |
|  |  | 234 | 5 |  | ND | 0.95238 | Safe | ND | Undetermined | Safe | 2.564 | 0.47619 | Contaminated |
|  |  |  |  |  |  |  |  |  |  |  |  |  |  |
| 53 | Rajbari | 235 | 2 | Pangsha | ND | 4.28571 | Safe | ND | Undetermined | Safe | ND | 0.71429 | Safe |
|  |  | 236 | 3 |  | 11.341 | 3.75 | Contaminated | ND | Undetermined | Safe | 2.156 | 2.5 | Safe |
|  |  | 237 | 5 |  | ND | 0.95238 | Safe | ND | Undetermined | Safe | ND | 0.47619 | Safe |
|  |  |  |  |  |  |  |  |  |  |  |  |  |  |
| 54 | Rajshahi | 238 | 1 | 2,3,4 = Motihar; 1,5 = Rajpara | 14.286 | 0.47619 | Contaminated | ND | Undetermined | Safe | 3.644 | 0.47619 | Contaminated |
|  |  | 239 | 2 |  | ND | 4.28571 | Safe | ND | Undetermined | Safe | 0.331 | 0.71429 | Safe |
|  |  | 240 | 3 |  | 6.6 | 3.75 | Contaminated | ND | Undetermined | Safe | 1.042 | 2.5 | Safe |
|  |  | 241 | 4 |  | ND | 2 | Safe | ND | Undetermined | Safe | 0.794 | 2 | Safe |
|  |  | 242 | 5 |  | ND | 0.95238 | Safe | 10.697 | 56.15925 | Safe | 0.339 | 0.47619 | Safe |
|  |  |  |  |  |  |  |  |  |  |  |  |  |  |
| 55 | Rangamati | 243 | 1 | Rangamati sadar | 8.202 | 0.47619 | Contaminated | ND | Undetermined | Safe | 5.484 | 0.47619 | Contaminated |
|  |  | 244 | 2 |  | ND | 4.28571 | Safe | ND | Undetermined | Safe | 0.92 | 0.71429 | Contaminated |
|  |  | 245 | 4 |  | ND | 2 | Safe | ND | Undetermined | Safe | ND | 2 | Safe |
|  |  | 246 | 5 |  | ND | 0.95238 | Safe | ND | Undetermined | Safe | 0.1 | 0.47619 | Safe |
|  |  |  |  |  |  |  |  |  |  |  |  |  |  |
| 56-1 | Rangpur 1 | 247 | 1 | Rangpur sadar | ND | 0.47619 | Safe | ND | Undetermined | Safe | - | - | - |
|  |  | 248 | 3 |  | ND | 3.75 | Safe | ND | Undetermined | Safe | ND | 2.5 | Safe |
|  |  | 249 | 4 |  | 29.273 | 2 | Contaminated | ND | Undetermined | Safe | 2.063 | 2 | Contaminated |
|  |  | 250 | 5 |  | ND | 0.95238 | Safe | ND | Undetermined | Safe | ND | 0.47619 | Safe |
|  |  |  |  |  |  |  |  |  |  |  |  |  |  |
| 56-2 | Rangpur 2 | 251 | 1 | Badargonj | 7.601 | 0.47619 | Contaminated | ND | Undetermined | Safe | 2.583 | 0.47619 | Contaminated |
|  |  | 252 | 2 |  | 0.147 | 4.28571 | Safe | ND | Undetermined | Safe | ND | 0.71429 | Safe |
|  |  | 253 | 3 |  | ND | 3.75 | Safe | ND | Undetermined | Safe | 1.533 | 2.5 | Safe |
|  |  | 254 | 4 |  | ND | 2 | Safe | ND | Undetermined | Safe | ND | 2 | Safe |
|  |  | 255 | 5 |  | ND | 0.95238 | Safe | ND | Undetermined | Safe | 0.192 | 0.47619 | Safe |
|  |  |  |  |  |  |  |  |  |  |  |  |  |  |
| 57 | Shariatpur | 256 | 2 | Jajira | ND | 4.28571 | Safe | ND | Undetermined | Safe | ND | 0.71429 | Safe |
|  |  | 257 | 3 |  | ND | 3.75 | Safe | ND | Undetermined | Safe | - | - | - |
|  |  | 258 | 4 |  | ND | 2 | Safe | ND | Undetermined | Safe | ND | 2 | Safe |
|  |  | 259 | 5 |  | 4.541 | 0.95238 | Contaminated | ND | Undetermined | Safe | 0.193 | 0.47619 | Safe |
|  |  |  |  |  |  |  |  |  |  |  |  |  |  |
| 58 | Satkhira | 260 | 1 | Kaligonj | ND | 0.47619 | Safe | ND | Undetermined | Safe | ND | 0.47619 | Safe |
|  |  | 261 | 2 |  | ND | 4.28571 | Safe | ND | Undetermined | Safe | 0.344 | 0.71429 | Safe |
|  |  | 262 | 3 |  | 0.386 | 3.75 | Safe | ND | Undetermined | Safe | 2.22 | 2.5 | Safe |
|  |  | 263 | 4 |  | ND | 2 | Safe | ND | Undetermined | Safe | 3.426 | 2 | Contaminated |
|  |  | 264 | 5 |  | ND | 0.95238 | Safe | ND | Undetermined | Safe | ND | 0.47619 | Safe |
|  |  |  |  |  |  |  |  |  |  |  |  |  |  |
| 59 | Sherpur | 265 | 1 | Sherpur sadar | ND | 0.47619 | Safe | ND | Undetermined | Safe | 0.619 | 0.47619 | Contaminated |
|  |  | 266 | 2 |  | ND | 4.28571 | Safe | ND | Undetermined | Safe | ND | 0.71429 | Safe |
|  |  | 267 | 3 |  | ND | 3.75 | Safe | ND | Undetermined | Safe | 5.323 | 2.5 | Contaminated |
|  |  | 268 | 4 |  | ND | 2 | Safe | ND | Undetermined | Safe | 0.897 | 2 | Safe |
|  |  | 269 | 5 |  | ND | 0.95238 | Safe | ND | Undetermined | Safe | ND | 0.47619 | Safe |
|  |  |  |  |  |  |  |  |  |  |  |  |  |  |
| 60 | Sirajganj | 270 | 1 | Shahjadpur | ND | 0.47619 | Safe | ND | Undetermined | Safe | 0.741 | 0.47619 | Contaminated |
|  |  | 271 | 2 |  | ND | 4.28571 | Safe | ND | Undetermined | Safe | 0.448 | 0.71429 | Safe |
|  |  | 272 | 3 |  | ND | 3.75 | Safe | ND | Undetermined | Safe | 1.299 | 2.5 | Safe |
|  |  | 273 | 4 |  | ND | 2 | Safe | ND | Undetermined | Safe | ND | 2 | Safe |
|  |  | 274 | 5 |  | ND | 0.95238 | Safe | ND | Undetermined | Safe | 0.095 | 0.47619 | Safe |
|  |  |  |  |  |  |  |  |  |  |  |  |  |  |
| 61 | Sunamganj | 275 | 1 | 1,3,4 = Sunamgonj Sadar; 2,5 = Bisomvorpur | 2.385 | 0.47619 | Contaminated | ND | Undetermined | Safe | 0.348 | 0.47619 | Safe |
|  |  | 276 | 2 |  | ND | 4.28571 | Safe | ND | Undetermined | Safe | 0.436 | 0.71429 | Safe |
|  |  | 277 | 3 |  | ND | 3.75 | Safe | ND | Undetermined | Safe | 0.886 | 2.5 | Safe |
|  |  | 278 | 4 |  | ND | 2 | Safe | ND | Undetermined | Safe | 0.591 | 2 | Safe |
|  |  | 279 | 5 |  | 2.44 | 0.95238 | Contaminated | ND | Undetermined | Safe | 0.244 | 0.47619 | Contaminated |
|  |  |  |  |  |  |  |  |  |  |  |  |  |  |
| 62 | Sylhet | 280 | 1 | Golapgonj | ND | 0.47619 | Safe | ND | Undetermined | Safe | ND | 0.47619 | Safe |
|  |  | 281 | 2 |  | 5.434 | 4.28571 | Contaminated | ND | Undetermined | Safe | 0.449 | 0.71429 | Safe |
|  |  | 282 | 3 |  | ND | 3.75 | Safe | ND | Undetermined | Safe | ND | 2.5 | Safe |
|  |  | 283 | 4 |  | 15.015 | 2 | Contaminated | ND | Undetermined | Safe | 1.276 | 2 | Safe |
|  |  | 284 | 5 |  | ND | 0.95238 | Safe | ND | Undetermined | Safe | 0.239 | 0.47619 | Safe |
|  |  |  |  |  |  |  |  |  |  |  |  |  |  |
| 63 | Tangail | 285 | 1 | Ghatail | 3.094 | 0.47619 | Contaminated | ND | Undetermined | Safe | ND | 0.47619 | Safe |
|  |  | 286 | 3 |  | 3.876 | 3.75 | Contaminated | ND | Undetermined | Safe | ND | 2.5 | Safe |
|  |  | 287 | 4 |  | ND | 2 | Safe | ND | Undetermined | Safe | 0.346 | 2 | Safe |
|  |  | 288 | 5 |  | ND | 0.95238 | Safe | ND | Undetermined | Safe | ND | 0.47619 | Safe |
|  |  |  |  |  |  |  |  |  |  |  |  |  |  |
| 64 | Thakurgaon | 289 | 1 | 1,2,5 = Ranisonkoil; 4 = Horipur | ND | 0.47619 | Safe | ND | Undetermined | Safe | 2.1 | 0.47619 | Contaminated |
|  |  | 290 | 2 |  | 6.467 | 4.28571 | Contaminated | ND | Undetermined | Safe | 1.11 | 0.71429 | Contaminated |
|  |  | 291 | 4 |  | ND | 2 | Safe | ND | Undetermined | Safe | ND | 2 | Safe |
|  |  | 292 | 5 |  | ND | 0.95238 | Safe | ND | Undetermined | Safe | ND | 0.47619 | Safe |

*ND = Not Detected (at ppb level for 0.2g dry sample digested into 100 ml volume), **DW = Dry Weight, ***FW = Fresh Weight

**Legend: Vegetable Serial**

1 – White Potato

2 – Green Cabbage

3 – Red Spinach

4 – White Radish

5 – Green Bean
